# A Molecular Phylogeny of the Lichen Genus *Lecidella* Focusing on Species from Mainland China

**DOI:** 10.1371/journal.pone.0139405

**Published:** 2015-09-28

**Authors:** Xin Zhao, Lu Lu Zhang, Zun Tian Zhao, Wei Cheng Wang, Steven D. Leavitt, Helge Thorsten Lumbsch

**Affiliations:** 1 College of Life Sciences, Shandong Normal University, Jinan, 250014, P. R. China; 2 Science & Education, The Field Museum, Chicago, Illinois, United States of America; 3 Committee on Evolutionary Biology, University of Chicago, Chicago, Illinois, United States of America; University of Florida, UNITED STATES

## Abstract

The phylogeny of *Lecidella* species is studied, based on a 7-locus data set using ML and Bayesian analyses. Phylogenetic relationships among 43 individuals representing 11 *Lecidella* species, mainly from mainland China, were included in the analyses and phenotypical characters studied and mapped onto the phylogeny. The *Lecidella* species fall into three major clades, which are proposed here as three informal groups–*Lecidella stigmatea* group, *L*. *elaeochroma* group and *L*. *enteroleucella* group, each of them strongly supported. Our phylogenetic analyses support traditional species delimitation based on morphological and chemical traits in most but not all cases. Individuals considered as belonging to the same species based on phenotypic characters were found to be paraphyletic, indicating that cryptic species might be hidden under these names (e.g. *L*. *carpathica* and *L*. *effugiens*). Potentially undescribed species were found within the phenotypically circumscribed species *L*. *elaeochroma* and *L*. *stigmatea*. Additional sampling across a broader taxonomic and geographic scale will be crucial to fully resolving the taxonomy in this cosmopolitan genus.

## Introduction

The circumscription of lichen-forming fungal species has traditionally been guided by morphological, chemical and ecological features. However, because lichens generally display few taxonomically useful characters, of which many are widely variable and the homology of character states within and among groups is difficult to assess [1–4]. Therefore, molecular data have gained importance in lichen systematics and now have a significant impact on the classification and taxonomy of lichenized ascomycetes [5–9]. Many phylogenetic studies of lichenized ascomycetes are designed to test morphology-based classifications. As a result, the systematic value of morphological characters in diverse groups of lichen-forming fungi is now much better understood [10–15]. Recent studies indicate that while phenotypical characters are useful in discriminating distinct lineages in many cases, they may fail to separate distinct species-level lineages with similar morphologies and/or chemistries [16–21]. In some cases, cryptic lineages correspond to geographically isolated areas, often continents, and are well documented in foliose or fruticose lichens. However, less is known about the presence and biogeography of cryptic lineages in crustose lichens.

The genus *Lecidella* Körb. (Lecanoraceae; [22]) is a medium-size genus with approximately 80 accepted species [23]. The species are distinguished based on a few taxonomically diagnostic morphological and anatomical characters, in addition to secondary chemistry. The genus is generally regarded as taxonomically difficult due to a high degree of variation and/or plasticity in diagnostic characters. As lecideoid lichens, *Lecidella* was considered to be part of the huge genus *Lecidea* Ach. for a long time [24]. Although *Lecidella* was established by Körber in 1855 [25], the genus was not widely used until Hertel [26] recognized it as a subgenus of *Lecidea* and subsequently [27] at generic level mainly based on the secondary chemistry which differs from *Lecidea* in the presence of chlorinated norlichexanthones. *Lecidella* was classified in Lecanoraceae [28, 29] mainly based on the similarity in ascus-type. Our knowledge of species delimitation in the genus vastly improved over the last decades with the improvement of identification of chlorinated xanthones using TLC (thin layer chromatography), HPLC (high performance liquid chromatography), and mass spectrometry [30–39]. As currently circumscribed, *Lecidella* is comprised of crustose lichens characterized by black lecideine apothecia, persistent proper excipulum, clavate, large amyloid, eight-spored asci referred to as *Lecidella*-type [29], paraphyses that typically separate readily in KOH, simple, hyaline, non-halonate ascospores, curved, filiform conidia and the occurrence of xanthones in the majority of species. The genus is widespread, from the tropics to polar regions, occurring on various substrata, including rock, detritus, bark, wood and mosses. Presently, about 10 species of *Lecidella* have been reported in mainland China [40–42].

Currently our knowledge of the phylogeny of *Lecidella* is limited, with only a few studies including representatives from this genus [43–49]. While these few studies support the monophyly of *Lecidella* and its placement in Lecanoraceae, none have included a broader taxonomic sampling to address the evolutionary relationships of species within the genus. Here we address the phylogeny of the genus using a concatenated data set of seven loci (two loci from the nuclear ribosomal cistron, one mitochondrial ribosomal, and four nuclear protein-coding genes) for selected taxa of *Lecidella*, with a focus on species from mainland China. The main goals of this study were 1) to evaluate the current delimitation of selected morphospecies within *Lecidella* and 2) to reconstruct phylogenetic relationships among species of this genus.

## Materials and Methods

### MorphologicAl and chemical studies

Approximately 1000 specimens of *Lecidella* collected in mainland China were studied primarily from the herbarium: SDNU, and also the herbaria: DUKE, F, HMAS-L, and KUN-L. The field studies did not involve endangered or protected species, and no specific permissions were required to collect specimens from these localities. Specimens were examined using standard microscopy techniques and hand-cut sections under a dissecting microscope (Nikon SMZ 745T), anatomical characters were observed under a compound microscope in water (OLYMPUS CX21). Secondary metabolites were identified using thin-layer chromatography with solvent C (toluene: acetic acid = 170:30) [50, 51]. Specimens representing the range of morphological and chemical variation were selected for molecular phylogenetic investigations.

### Taxon sampling and molecular data

For our phylogenetic analyses, we selected a total of 51 *Lecidella* specimens collected from different regions of mainland China. These specimens represented 11 currently recognized species–*L*. *carpathica*, *L*. *effugiens*, *L*. *elaeochroma*, *L*. aff. *elaeochroma*, *L*. *elaeochromoides*, *L*. *enteroleucella*, *L*. *euphorea*, *L*. aff. *euphorea*, *L*. *patavina*, *L*. *stigmatea* and *L*. *tumidula* (S1 Table). Each species was represented by at least two specimens. Molecular data were generated for seven loci: the internal transcribed spacer (ITS), nuclear large subunit (nucLSU), mitochondrial small subunit (mtSSU), minichromosome maintenance complex component 7 (MCM7), ribosome biogenesis gene (TSR1), the largest subunit of the RNA polymerase II gene (RPB1) and the second largest subunit of RNA polymerase II gene (RPB2). Sequences generated for this study were complemented with sequences from GenBank representing additional specimens or species: *L*. *elaeochroma*, *L*. *euphorea*, *L*. *flavosorediate*, *L*. *greenii*, *L*. *meiococca*, *L*. *siplei*, *L*. *stigmatea and L*. *wulfenii* (S2 Table). *Rhizoplaca parilis*, *R*. *porterii*, *Protoparmeliopsis achariana* and *P*. *muralis* were selected as outgroups (S2 Table).

To obtain fungal sequences apothecia were used for extracting total genomic DNA using the Prepease DNA Isolation Kit (USB, Cleveland, OH, USA), following the leaf extraction protocol. Ready-To-Go PCR Beads were used (GE Healthcare) for the amplification of the targeted loci. A new forward primer for amplifying the RPB2 locus of *Lecidella* was designed for this study, and all primers and PCR settings [52–63] are summarized in Table 1. PCR products were purified using Exo SAP-IT (USB, Cleveland, OH, USA), following the manufacturers’ instructions. Sequencing reactions were performed using BigDye Terminator v3.1 (Applied Biosystems, Foster City, CA,USA) and using the same primers as those used for amplification. Sequenced products were then run on an ABI 3730 automated sequencer according to established protocols (Applied Biosystems) at the Pritzker Laboratory for Molecular Systematics at the Field Museum, Chicago, IL, USA.

**Table 1:** Primer information and PCR settings used for this paper.

Loci/PCR info	ITS	nucLSU	mtSSU	MCM7	RPB1	RPB2	TSR1
PCR primers	ITS1f^1^	AL1R^3^	mrSSU1^6^	LecMCM7f^8^	gRPB1a^10^	*Lecidella*_RPB2f	TSR1f (5’-GCTTGAGRACRAGICADTGGGAGAC-3’)
	ITS4a^2^	AL2R^4^	mrSSU2r^6^	LecMCM7r^8^	fRPB1c^11^	(5’-DGATGTGCGAGAYCGAGART-3’)	TSR1r (5’-CATRTAICKRAIRGTGACVAGCTTC-3’)
		LR5^5^	mrSSU3r^6^	MCM7-709f^9^		RPB2-6f^12^	
		LR6^5^	MSU1^7^	MCM7-1348r^9^		RPB2-7cr^12^	
			MSU7^7^				
Initial denaturation	95°C 5min	95°C 5min	94°C 10min	94°C 10min	94°C 10min	94°C 10min	94°C 10min
Phase 1	10 cycles	10 cycles	34 cycles	34 cycles	34 cycles	34 cycles	34 cycles
	95°C 30sec	95°C 30sec	95°C 45sec	94°C 45sec	94°C 45sec	94°C 45sec	94°C 45sec
	66°C 30sec	66°C 30sec	50°C 45sec	50°C 50sec	50°C 50sec	50°C 50sec	50°C 50sec
	72°C 1min30sec	72°C 1min30sec	72°C 1min30sec	72°C 1min	72°C 1min	72°C 1min	72°C 1min
Phase 2	34 cycles	34 cycles	none	none	none	none	none
	95°C 30sec	95°C 30sec					
	56°C 30sec	56°C 30sec					
	72°C 1min30sec	72°C 1min30sec					
Final extension	72°C 10min	72°C 10min	72°C 10min	72°C 5min	72°C 5min	72°C 5min	72°C 5min

^1^ [52]

^2^ [53]

^3^ [54]

^4^ [55]

^5^ [56]

^6^ [57]

^7^ [58]

^8^ [59]

^9^ [60]

^10^ [61]

^11^ [62]

^12^ [63].

### Sequence alignments

Contigs were assembled and edited using the program Geneious v6.1.2 (Biomatters Ltd., Auckland, NZ). Sequences of each locus were aligned using the program MAFFT v7 [64]. For ITS sequences, we used the L-ING-i alignment algorithm with the remaining parameters set to default values. For nucLSU, G-ING-i algorithm and “leave gappy regions” were selected. Then we used E-ING-i algorithm for mtSSU and RPB1, and G-ING-i algorithm for MCM7, TSR1 and RPB2, with the remaining parameters set to default values. Ambiguous positions of the ITS and mtSSU alignments were removed using Gblocks [65] implementing all the options for a less stringent selection. We compiled two data matrices: the ‘ITS matrix’ included ITS sequences of all of our samples (Table 2) and all taxa of *Lecidella* available from GenBank (S2 Table); and the ‘multilocus matrix’ was a concatenated dataset comprised of specimens that were represented by at least two of seven targeted loci (Table 2). A summary of alignment information for the multilocus dataset was provided (Table 3). The ‘multilocus matrix’ included a total of 47 individuals representing 16 taxa.

**Table 2:** Species and sequences used for the multilocus phylogenetic analyses, newly generated sequences are in bold. The numbers behind the specific epithet are used to distinguish different individuals for each species.

Species	ITS	LSU	mtSSU	RPB1	RPB2	MCM7	TSR1
*Lecidella* aff.*elaeochroma* 0	KT453753	KT453778	KT453825		KT453961	KT453884	**KT453980**
*Lecidella* aff.*elaeochroma* 1	KT453752	KT453779	KT453826		KT453962	KT453885	**KT453981**
*Lecidella* aff.*elaeochroma* 2	KT453751	KT453780	KT453827		KT453963		
*Lecidella* aff.*euphorea* 0	KT453756	**KT453781**	KT453828		**KT453960**		
*Lecidella* aff.*euphorea* 5	**KT453755**	**KT453782**	**KT453829**		**KT453959**		
*Lecidella carpathica* 0	**KT453739**	**KT453783**	**KT453830**		**KT453942**		
*Lecidella carpathica* 1	KT453741	KT453784	KT453831	KT453905	KT453944		
*Lecidella carpathica* 2	**KT453740**				**KT453955**		
*Lecidella carpathica* 3	KT453738				**KT453943**		
*Lecidella effugiens* 0	KT453748	KT453785	KT453832		KT453949		
*Lecidella effugiens* 1	KT453747	KT453786	KT453833		KT453941	KT453883	
*Lecidella effugiens* 2		**KT453787**			**KT453957**		
*Lecidella effugiens* 3	**KT453754**	**KT453788**	**KT453834**		**KT453958**		
*Lecidella elaeochroma* 3	KT453749	**KT453789**	KT453835		**KT453956**		**KT453984**
*Lecidella elaeochroma* 5	HQ650605	DQ986747		DQ986818	DQ992429		
*Lecidella elaeochromoides* 0	KT453750	KT453790	KT453836		KT453940		
*Lecidella elaeochromoides* 1	KT453746				**KT453948**		
*Lecidella enteroleucella* 0		**KT453791**	**KT453837**		**KT453963**		
*Lecidella enteroleucella* 1	KT453757	KT453792	KT453838		KT453965		
*Lecidella enteroleucella* 2		**KT453793**	**KT453839**		**KT453966**		
*Lecidella euphorea* 1		**KT453794**	**KT453840**		**KT453953**		
*Lecidella euphorea* 2	**KT453744**		**KT453841**		**KT453951**		
*Lecidella euphorea* 3	**KT453743**	**KT453795**			**KT453952**		
*Lecidella euphorea* 4		**KT453796**	**KT453842**		**KT453954**		
*Lecidella euphorea* 7	KT453745	**KT453797**	KT453843	**KT453908**	**KT453947**		
*Lecidella euphorea* 8	**KT453742**	**KT453798**	**KT453844**	**KT453909**	**KT453950**		
*Lecidella meiococca*	AF517929	AY300842	AY300893				
*Lecidella patavina* 0	KT453767	KT453799	KT453845	KT453910	KT453967	KT453879	
*Lecidella patavina* 1		KT453800	KT453846	KT453911	KT453968		
*Lecidella patavina* 2	**KT453761**	**KT453801**					
*Lecidella stigmatea* 0	KT453762		KT453847	KT453912	KT453969		
*Lecidella stigmatea* 1	KT453760	KT453802	KT453848	KT453914	KT453970	KT453880	
*Lecidella stigmatea* 2	KT453766	KT453803	KT453849	KT453918	KT453971		**KT453985**
*Lecidella stigmatea* 3	KT453764	KT453804		KT453915	KT453974		
*Lecidella stigmatea* 4	**KT453768**	**KT453805**			**KT453977**		
*Lecidella stigmatea* 5	KT453765	KT453806	KT453850	KT453916	KT453976	KT453881	**KT453987**
*Lecidella stigmatea* 6	**KT453759**				**KT453975**		
*Lecidella stigmatea* 7	KT453763	KT453807	KT453851	KT453917	KT453973	KT453882	**KT453986**
*Lecidella stigmatea* 8	KT453758	KT453808	KT453852	KT453913	KT453972		
*Lecidella stigmatea* 9		KJ766590		KJ766866			
*Lecidella tumidula* 1	KT453737	KT453809	KT453853	KT453906	KT453945		**KT453982**
*Lecidella tumidula* 2	KT453736	KT453810	KT453854	KT453907	KT453946		**KT453983**
*Lecidella tumidula* 3	HQ650596		DQ986784	DQ986857	DQ992479		
*Protoparmeliopsis achariana* 0		DQ973027	DQ972976	DQ973051	DQ973088		
*Protoparmeliopsis muralis*		KJ766634	KJ766466	KJ766830	KJ766943		
*Rhizoplaca parilis*	HM577309	KT453814	KT453859	JX948311	JX948350	HM577443	
*Rhizoplaca porterii*	HM577376	KT453816	KT453861	JX948340	JX948379	HM577510	

**Table 3:** The alignment information for the multilocus dataset.

Alignments	ITS	nucLSU	mtSSU	MCM7	RPB1	RPB2	TSR1	Total
Number of sequences	38	40	36	9	21	44	8	196
Newly added sequences to Genbank	10	15	9	0	2	19	8	64
Number of sites (including gaps)	509	696	595	502	679	656	623	4260
Missing sequences/ the percentages	9/19%	7/15%	11/23%	38/81%	26/55%	3/6%	39/83%	133/40%
Nucleotide substitution models	GTR+I+G	SYM+G	GTR+I+G	HKY+I+G	GTR+I+G	SYM+I+G	HKY+G	

### Phylogenetic analysis

The single-locus alignments were concatenated in Geneious for the subsequent phylogenetic analyses. Maximum likelihood (ML) analyses were carried out on the ‘multilocus matrix’ using locus-specific model partitions (ITS, nucLSU, mtSSU, MCM7, RPB1, RPB2 and TSR1) in RAxML v8.1.11 [66]. We were unable to generated MCM7 and TSR1 sequences from the majority of specimens, and therefore we did another partitioned ML analyses limited to the five most thoroughly sampled loci (ITS, nucLSU, mtSSU, RPB1 and RPB2). A search combining 200 separate ML searches was conducted, implementing a GTRGAMMA model, and 1000 pseudoreplicates to evaluate bootstrap support for each node. A ML topology was also inferred from the ‘ITS matrix’ using RAxML and search parameters as described above. In addition to the ML analysis, the ‘multilocus matrix’ was also subjected to a Bayesian analysis with MrBayes v3.2.3 [67]. The nucleotide substitution models of the seven loci were selected using the Akaike information criterion in jModelTest v2.1.7 [68]. The Bayesian analysis was run for 10,000,000 generations with four independent chains and sampling every 1000th tree. All model parameters were unlinked. Two independent Bayesian runs were conducted to ensure that stationarity was reached and the runs converged at the same log-likelihood level (verified by eye and with AWTY option; [69]). After discarding the burn-in, the remaining 7500 trees of each run were pooled to calculate a 50% majority rule consensus tree. The clades that received bootstrap support ≥70% under ML and posterior probabilities ≥0.95 were considered significant. Phylogenetic trees were visualized using FigTree v1.4.2 [70].

## Results

**Fig 1:**
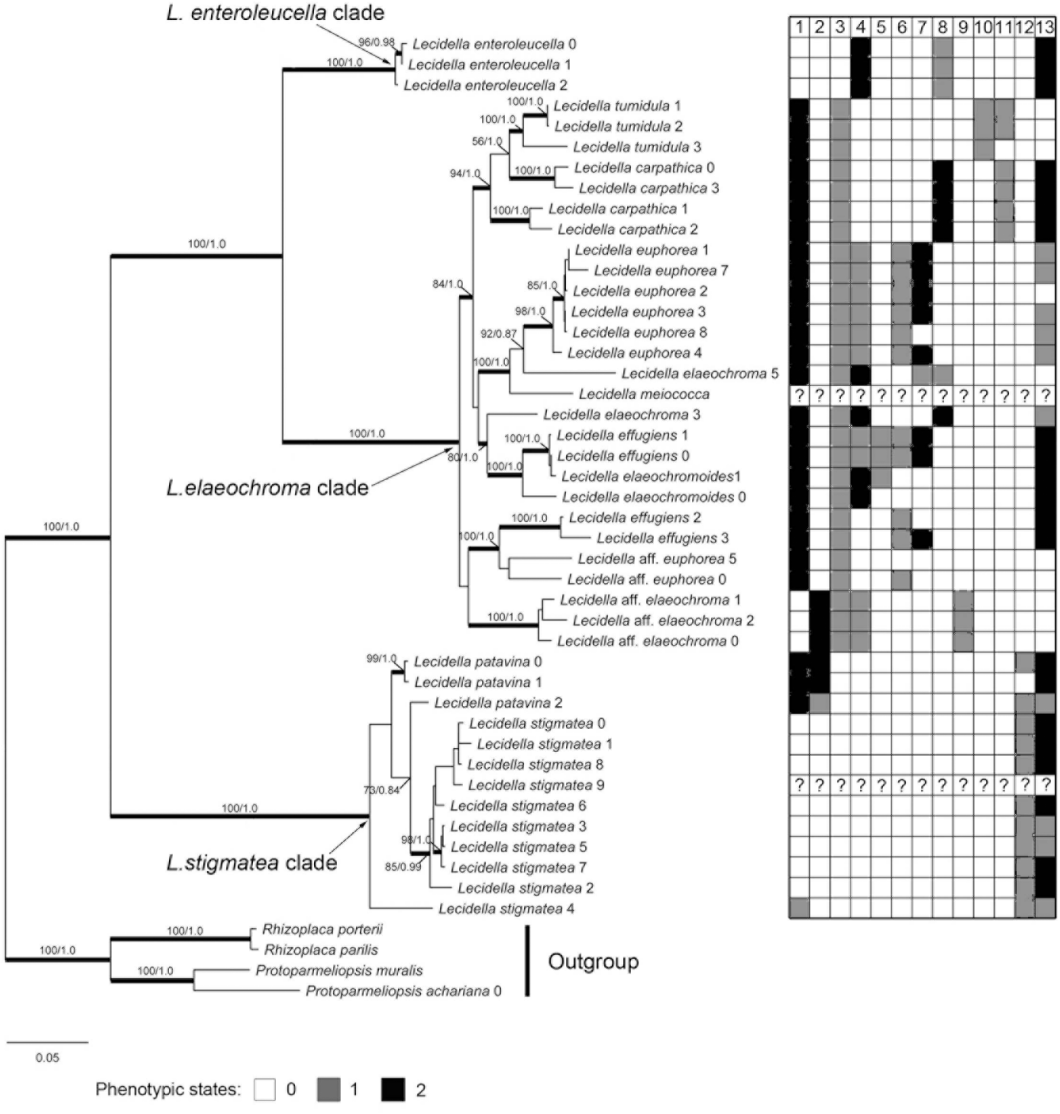
Maximum Likelihood phylogeny of *Lecidella* inferred from a concatenated 7-locus data matrix. Branches in bold received maximum likelihood bootstrap support values equal or above 70% and posterior probabilities equal or above 0.95. Phenotypical characters listed in boxes right to the tree. 1. Color of epihymenium: 0 = olive-brown (sometimes with green tinge); 1 = violet-brown; 2 = green to dark-green. 2. Hymenium: 0 = not inspersed; 1 = slightly inspersed; 2 = inspersed. 3. Color of hypothecium: 0 = colorless; 1 = yellow-brown to red-brown. 4. Arthothelin: 0 = absent; 1 = present as minor substance; 2 = present as a major substance. 5. Thiophanic acid: 0 = absent; 1 = present. 6. Aotearone: 0 = absent; 1 = present. 7. Capistratone: 0 = absent; 1 = present as minor substance; 2 = present as major substance. 8. Thuringione: 0 = absent; 1 = present as minor substance; 2 = present as major substance. 9. Granulosin: 0 = absent; 1 = present. 10. Lichexanthone: 0 = absent; 1 = present. 11. Diploicin: 0 = absent; 1 = present. 12. Zeorin: 0 = absent; 1 = present. 13. Substratum: 0 = corticolous; 1 = lignicolous; 2 = saxicolous. (? indicates samples that were unavailable for study)

For this study, 63 new sequences were generated (Table 2). The two datasets we used in this study were deposited in TreeBase (ID #17997). The ‘ITS matrix’ consisted of 60 individuals and 550 aligned nuclecotide position characters. The concatenated, ‘multilocus matrix’ consisted of 47 individuals and 4260 aligned nuclecotide position characters (Table 3). Phylogenies derived from the ML and B/MCMC analyses were generally concordant. ML analysis of the seven-locus and five-locus matrices also produced topologies that were generally concordant, and the seven-locus phylogeny is presented here, with nodal support values from both ML bootstrap analysis and posterior probabilities from the Bayesian inference (Fig 1). We listed 13 phenotypical characters next to the ML tree (Fig 1). The phylogenies resulting from the ML analyses of the ITS matrix and the five-locus matrix were shown in S1 and S2 Figs.

The most likely tree was comprised of three main, well-supported (bs = 100%, pp = 1.0) clades (Fig 1). *Lecidella enteroleucella* formed a highly supported clade (bs = 100%, pp = 1.0) sister to *L*. *carpathica*, *L*. *effugiens*, *L*. *elaeochroma*, *L*. aff. *elaeochroma*, *L*. *elaeochromoides*, *L*. *euphorea*, *L*. aff. *euphorea*, *L*. *meiococca*, and *L*. *tumidula*, which together formed a well-supported clade (bs = 100%, pp = 1.0). The species *L*. *stigmatea* and *L*. *patavina* formed a well-supported sister-group (bs = 100%, pp = 1.0) to the other two clades. All species with colorless hypothecium and lacking xanthones, which refer to *L*. *stigmatea* and *L*. *patavina* in the seven-gene tree (Fig 1) and *L*. *greenii*, *L*. *siplei*, *L*. *stigmatea* and *L*. *patavina* in the ITS tree (S1 Fig) formed a monophyletic lineage. *Lecidella enteroleucella*, which is characterized by a colorless hypothecium and contains the chlorinated xanthones thuringione and arthothelin, formed a separate clade. While most species which have yellow-brown to brown hypothecium and contain xanthones formed a separate clade, the traditionally circumscribed species *L*. *carpathica* was not recovered as a monophyletic lineage but clustered with *L*. *tumidula* in both trees.

## Discussion

Here we provide the first phylogenetic hypothesis for the cosmopolitan lichen-forming fungal genus *Lecidella*. In most cases, our phylogenetic analyses support the traditional species delimitation based on morphological and chemical traits. The phylogeny also indicated that the species diversity of *Lecidella* in China is higher than previously assumed. The role of secondary metabolites in species delimitation of the genus *Lecidella* is supported. Below we discuss each of the three major *Lecidella* clades recovered in this study.

### *Lecidella stigmatea* clade

This clade includes *L*. *stigmatea* and *L*. *patavina* and is characterized by a colorless hypothecium and secondary compounds other than xanthones. The two species were distinguished previously on a combination of characters, the inspersion of the hymenium being the most important [31]. Our phylogeny does not support the hymenial inspersion as being a major trait to characterize clades in the group. All *L*. *stigmatea* specimens collected in China have an olive-brown to brown (sometimes with green tinge) epihymenium regardless of the age of apothecia, while the samples identified as *L*. *patavina* have a green to dark-green epihymenium. In the ITS phylogeny (S1 Fig), *L*. *patavina* and *L*. *stigmatea* do not form monophyletic groups either and this indicates that the species circumscription in this group needs re-evaluation. Also, *L*. *greenii* is nested within the clade but forms a strongly supported monophyletic group. In the ITS tree (S1 Fig) the specimen ‘*L*. *patavina*2’ clusters with the Antarctic species *L*. *siplei* sensu Inoue [48, 71] and is also morphologically similar. With the data at hand we cannot conclude whether cryptic species are present in this group or whether the species are morphologically and chemically variable. However, the presence of well-supported groups within the clade suggests that cryptic species might be hidden under the names *L*. *patavina* and *L*. *stigmatea*. Our taxon sampling is insufficient to address this issue. The lignicolous sample ‘*L*. *stigmatea*4’ differs from the other *L*. *stigmatea* specimens in having a violet-brown epihymenium and thinner proper excipulum but otherwise agrees well morphologically with the saxicolous specimens of that species. In the ITS tree (S1 Fig) it is also separate from the other samples in this clade. Additional material will be necessary to determine whether it represents an undescribed taxon.

### *Lecidella elaeochroma* clade

This clade forms a well-supported monophyletic group in the phylogenies inferred from the ‘multilocus matrix’ (bs = 100%, pp = 1.0) and ‘ITS matrix’ (bs = 100%) and contains *L*. *carpathica*, *L*. *effugiens*, *L*. *elaeochroma*, *L*. aff. *elaeochroma*, *L*. *elaeochromoides*, *L*. *euphorea*, *L*. aff. *euphorea*, *L*. *meiococca*, and *L*. *tumidula*. In the ITS topology, *L*. *wulfenii* and *L*. *flavosorediata* were also recovered within this clade. All species within this clade are known to produce xanthones. According to Leuckert and Knoph [35] the xanthones in *Lecidella* mainly consist of two types: chloronorlichexanthones (arthothelin and thiophanic acid in our samples) and *O*-methylated xanthones (aotearone, capistratone, thuringione, granulosin and lichexanthone in our samples). Our results indicate that both major groups of xanthones are not completely independent from one another, being present for example in *L*. *euphorea*, which contains arthothelin and thiophanic acid but also aotearone and capistratone. *Lecidella euphorea* and *L*. *tumidula* form well-supported monophyletic clades. *Lecidella tumidula*3 was originally identified as *L*. *euphorea* [72]. However, a re-examination of the voucher specimen from DUKE revealed the presence of lichexanthone in this specimen; hence it was re-identified as *L*. *tumidula*. *Lecidella carpathica* is paraphyletic with *L*. *tumidula* nested within. In the ITS tree (S1 Fig), specimens *Lecidella* ‘*carpathica*1’ and ‘*carpathica*2’ cluster with a sequence from Genbank that was collected in Austria and apparently represents *L*. *carpathica* s.str., whereas specimens ‘*carpathica*0’ and ‘*carpathica*3’ form a sister-group to *L*. *tumidula* and potentially represent a cryptic lineage.

In our tree, *L*. *elaeochroma* is polyphyletic. A specimen from Xinjiang in NW China named *L*. ‘*elaeochroma*3’ did not cluster with *L*. ‘*elaeochroma*5’, which was collected in Europe (Belgium). The latter clustered in the ITS tree with numerous other sequences of *L*. *elaeochroma* and probably represents *L*. *elaeochroma* s. str., whereas the Chinese sample appear to represent an undescribed species. In addition, three samples were identified as *Lecidella* aff. *elaeochroma* here. They were collected in NE China (Jilin province) and have a brownish epihymenium (vs. greenish), a heavily inspersed hymenium (vs. non- to slightly inspersed), and a violet pigment in the excipulum. The samples also formed a separate, strongly supported monophyletic clade and might represent a new species (Fig 1). *Lecidella elaeochromoides* and *L*. *effugiens*, which were traditionally separated based on a combination of somewhat overlapping thalline and apothecial characters, and differences in chemical composition [33, 34], were not supported as distinct clades, suggesting these taxa may be conspecific. However, *L*. *effugiens* is not monophyletic and itself consist of two clades with two specimens (‘*effugiens*2’ and ‘*effugiens*3’) collected in NE and SW China potentially representing a cryptic lineage that is sister to *L*. aff. *euphorea* (Fig 1). The latter is only distantly related to *L*. *euphorea* but is morphologically similar. It, however, differs in its secondary chemistry, containing aotearone but lacking capistratone or lacking xanthones altogether (specimen 5).

### *Lecidella enteroleucella* clade

*Lecidella enteroleucella* individually forms a monophyletic group. The small apothecia (less than 0.4mm in diam.) are unique in the genus. The species agrees with the *L*. *elaeochroma* clade in containing chlorinated xanthones. In our tree the species forms a sister-group to that clade and no close relatives are known. However, *L*. *oceanica*, which was reported from Korea [73] and China [74], is morphologically similar but has a different chemistry (containing capistratone as major substance). We failed to generate DNA sequences of this species but hypothesize that *L*. *oceanica* also falls within this clade.

The current study provides important insight into the evolution and classification of *Lecidella*. Combining morphology, chemistry and phylogeny, the three informal groups within *Lecidella–L*. *elaeochroma* group, *L*. *enteroleucella* group and *L*. *stigmatea* group are proposed in this study. Additional sampling across a broader taxonomic and geographic scale will be crucial to fully resolving taxonomy in this cosmopolitan lineage.

## Supporting Information

S1 Fig Maximum Likelihood topology of *Lecidella* inferred from ITS sequences. ML bootstrap frequencies are shown above branches.
(TIF)

S2 Fig Maximum Likelihood topology of *Lecidella* inferred from the five-locus matrix. ML bootstrap frequencies are shown above branches.
(PDF)

S1 Table The detailed collection information for the *Lecidella* specimens selected for this study. (XLS)

S2 Table Species and specimens which were selected from GenBank, together with information on their origin and GenBank accession numbers. (XLS)
