# Supplementary figures and images for: A Molecular Phylogeny of the Lichen Genus Lecidella Focusing on Species from Mainland China
Source: PLoS One. 2015 Sep 28;10(9):e0139405. doi: 10.1371/journal.pone.0139405 (PMC4586381; doi:10.1371/journal.pone.0139405)

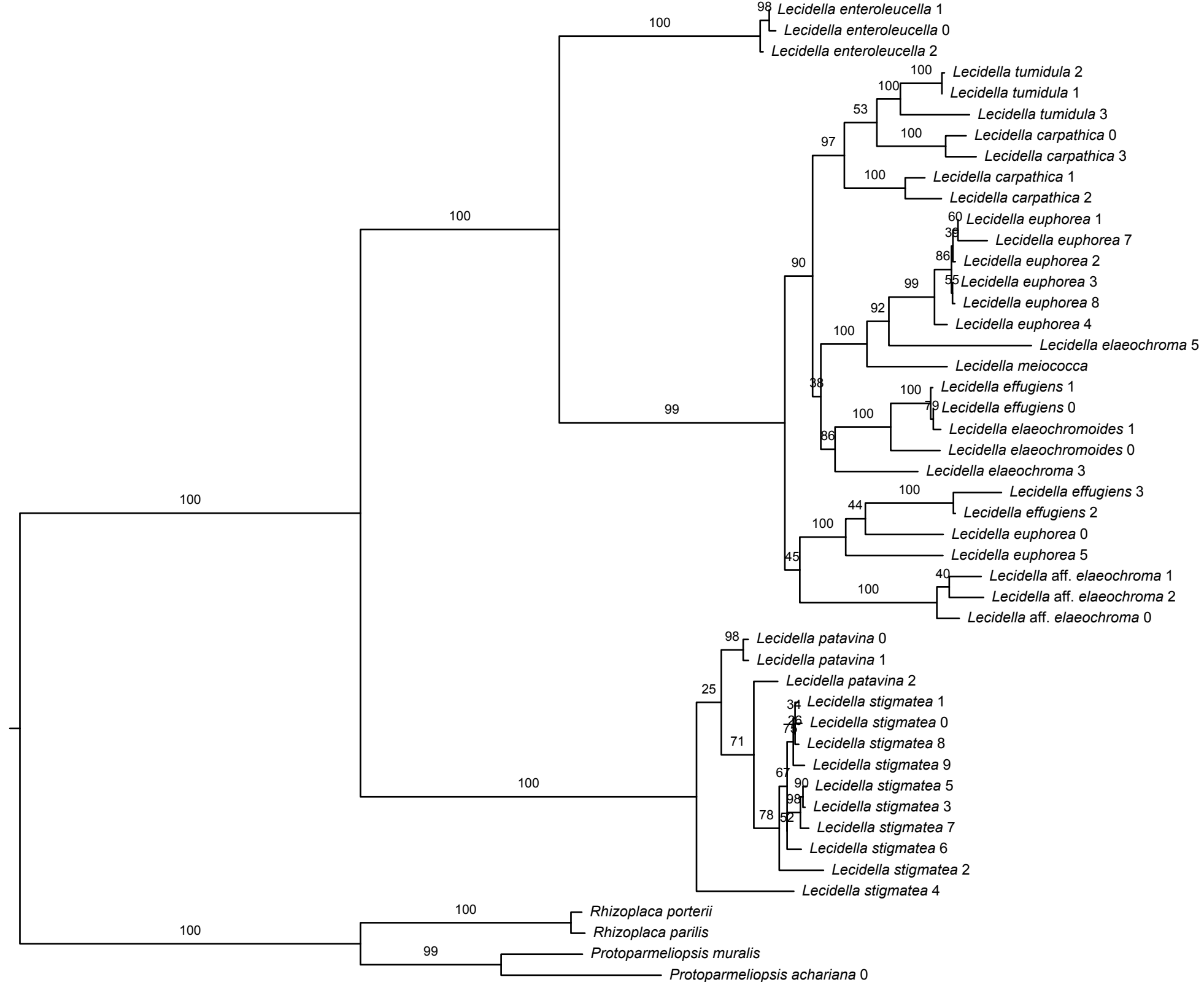

0.04

Supplement: S2 Fig — ML bootstrap frequencies are shown above branches. (PDF) [file pone.0139405.s002.pdf]
